# How much Fear? Exploring the Role of Integral Emotions on Stated Preferences for Wildlife Conservation

**DOI:** 10.1007/s00267-022-01593-z

**Published:** 2022-01-15

**Authors:** Sandra Notaro, Gianluca Grilli

**Affiliations:** 1 Department of Economics and Management, University of Trento, Via Inama 5/I, 38122 Trento, Italy; 2 Economic and Social Research Institute, Sir John Rogerson’s Quay, D02 Dublin, Ireland; 3 Trinity College Dublin, School of Social Sciences and Philosophy Dublin, Dublin, Ireland

**Keywords:** Human-wildlife interactions ∙ Emotional choices ∙ External stimuli ∙ Mass media communications ∙ Discrete Choice Experiments ∙ Context-dependence

## Abstract

Scientific evidence suggests that emotions affect actual human decision-making, particularly in highly emotionally situations such as human-wildlife interactions. In this study we assess the role of fear on preferences for wildlife conservation, using a discrete choice experiment. The sample was split into two treatment groups and a control. In the treatment groups the emotion of fear towards wildlife was manipulated using two different pictures of a wolf, one fearful and one reassuring, which were presented to respondents during the experiment. Results were different for the two treatments. The assurance treatment lead to higher preferences and willingness to pay for the wolf, compared to the fear treatment and the control, for several population sizes. On the other hand, the impact of the fear treatment was lower than expected and only significant for large populations of wolves, in excess of 50 specimen. Overall, the study suggests that emotional choices may represent a source of concern for the assessment of stable preferences. The impact of emotional choices is likely to be greater in situations where a wildlife-related topic is highly emphasized, positively or negatively, by social networks, mass media, and opinion leaders. When stated preferences towards wildlife are affected by the emotional state of fear due to contextual external stimuli, welfare analysis does not reflect stable individual preferences and may lead to sub-optimal conservation policies. Therefore, while more research is recommended for a more accurate assessment, it is advised to control the decision context during surveys for potential emotional choices.

## Introduction

The aim of the Convention on Biological Diversity (CBD 2010) to halting biodiversity loss was followed by policies and management actions to protect species worldwide. While costs of such policies and actions are clearly identified, their benefits are difficult to determine. Therefore, assessing the value of biodiversity preservation becomes important to compare costs and benefits of policies and management measures under limited financial resources (Rudd et al. 2016). This knowledge can inform conservation managers and policy makers about the value of endangered species to society and the economic incentives for their conservation. Stated preference methods are widely used in monetary valuations of biodiversity (Perrings and Kinzig 2021). Among these methods, the Discrete Choice Experiment (DCE) is particularly useful in providing information to policy makers and natural resources managers, since it allows distinguishing the relative preferences for different attributes and levels (Cerda et al. 2018), explicitly recognising the existence of trade-offs in decision making. However, when valuing environmental goods with stated preferences the level of willingness to pay (WTP) might be influenced by many factors. Previously, the role of socio-demographic characteristics on WTP levels, such as gender, age, education and income, knowledge of the non-market good and individual experience as well as framing or survey-design effects has been highlighted (Johnston et al. 2017). All these observable characteristics of the individual and survey elements have been proved to play a role in preference formation and in the explanation of individual choices.

Recently, scientific attention has been shifted to individual emotions. In the behavioural and psychological literature there is evidence that emotions affect the individual decision-making process (Blanchette and Richards 2010, Lerner et al. 2015), whereas it is unclear whether the same effect applies to stated preferences for public and environmental goods. As suggested by Hanley et al. (2017), the issue of stated preferences changing due to changing emotions is relevant, as it adds an element of context-dependence in field surveys. In fact, monetary valuations of the environment are based on the assumptions that (1) individuals make rational choices and that (2) preferences are stable and consistent (Hanley and Barbier 2009). When individual make choices based on contingent emotions, the assumptions of stability and consistency of their preferences are violated and welfare measurements are biased. Therefore, incorrect policy recommendations are communicated to decision-makers.

Few papers have investigated the effect of emotions on stated preferences and all focus on incidental emotions, i.e. emotions unrelated to the outcome of the decision at hand (Lerner et al. 2015). In a Contingent Valuation field experiment, Araña and León (2008) found a relevant and coherent impact of emotions on preferences for a network of trails in the island of Gran Canaria, Spain. In a DCE study for assessing a reduction in the environmental impact of a stone mining facility in the city of Las Palmas (Araña and León 2009), they showed that deviations from the compensatory decision rule^1^ was more frequent for individuals with a high emotional state. They also investigated the effects of induced sadness and disgust in a laboratory setting and found a heterogeneous role in compensatory rules. Hanley et al. (2017) found no impact on estimated parameters and WTP of induced happiness and sadness in a lab experiment, in which students were asked to express beach recreation preferences in New Zealand. Finally, Notaro et al. (2019) detected a significant impact on tourists’ preferences and WTP for the Alpine landscape in a field DCE survey. The existing literature on emotional state at the time of stated preference data collection is therefore insufficient to draw meaningful conclusions.

With this in mind, this paper investigates the association between emotions and stated preferences for wildlife conservation. While previous works concentrated on incidental emotions, a novel aspect of this work is the analysis of integral emotions, i.e. emotions arising from a decision at hand (Lerner et al. 2005).

The association between integral emotional states and stated choices is investigated. Integral emotional states represent an element of context-dependence in preference formation that violates the assumption of preference stability and may bias welfare analysis. This issue is particularly important in the context of wildlife conservation because it has been suggested that emotions together with value orientations (Freeman et al. 2021, Straka et al. 2020) impact decisions about wildlife (Hudenko 2012). Despite this, empirical studies on emotions towards wildlife concerning human-wildlife relationships are still scarce (Jacobs and Vaske, 2019). In particular, to the best of our knowledge, only Johansson et al. 2012 analyzed the relation among self-reported fear of encountering large carnivores and WTP for protection policies estimated with a Contingent Valuation study.

Emotions towards wild animals evolved over time (Castillo-Huitrón et al. 2020), and depending on the species can result in either positive or negative emotions (York and Longo 2017). Negative emotions, such as fear and disgust may generate negative attitudes towards some species, whereas positive emotions, such as happiness and joy, may generate positive attitudes for wildlife conservation (Jacobs 2012). However, emotions and attitudes towards wildlife can be altered by contextual situations. A drastic event involving a specific species can affect them, but massive media communication can also have an important effect on emotions and attitudes (Røskaft et al. 2003, Wieczorek 2012). Mass media provide wildlife representations that can be perceived positively or negatively by individuals, affecting thoughts, attitudes, emotions and behaviour (Potter 2012, Bombieri et al. 2018). For instance, extensive information on predators’ dangers may increase people’s negative attitudes *via* the mediating role of emotions, whereas emphasizing the positive appearances of wild animals, for example cute bears or wolf pups, may increase positive attitudes. Hughes et al. (2020) found that in North America the mass media over-report human-bear conflicts over scientific findings or positive stories about human-bear interactions. Journalists tend to frame stories for readers’ emotional response, dramatizing events and encounters, possibly influencing the public perceptions of bears. On the other hand, nature documentaries such as Our Planet tend to maintain the positive view of nature, generating positive attitudes toward the species (Jones et al. 2019). When stated preferences towards wildlife are affected by emotional states due to contextual external stimuli, welfare analysis does not reflect stable individual preferences and may lead to sub-optimal conservation policies.

To our knowledge, this is the first study that analyses willingness to pay for wildlife conservation in relation to fear due to external stimuli using a Discrete Choice Experiment (Bateman et al. 2002, Louviere et al. 2000), which is a knowledge gap in the literature. Furthermore, the analysis of field (face-to-face) survey data in which emotions are manipulated using pictures represents another novelty of this study and it is relevant, because field surveys offer lower control on respondents’ answers compared to lab studies.

The choice experiment investigated preferences for varying sizes of populations for wolves (*Canis lupus*), alpine lynx (*Lynx lynx*) and a subspecies of salamander (*Salamandra atra aurorae*) in a case study in the Italian Alps. While emotions towards all three animals were collected, treatments concentrated only on the emotion of fear for wolves. Wolf protection is highly dependent on a number of economic and social factors (Votsi et al. 2016), and emotions can play a decisive role in shaping social factors (Straka et al., 2020). We examined whether a fearful stimulus - a pictures of a snarling wolf - or a reassurance stimulus – a picture of a cheerful wolf - affected the level of fear towards wolves and preferences and WTP for wolf conservation. Our hypothesis is that the scary picture of a wolf may produce an increase in the level of fear for wolves and a decrease in the level of preference and willingness to pay for their conservation. On the contrary, the effect of the reassuring picture may be a decrease in the level of fear and an increase in preference and willingness to pay for wolf conservation.

The rest of paper is organized as follows. Next, we show a review of the literature on emotions and decision making, with a focus on wildlife conservation. Then, we present our methodology and the results obtained. Finally, we discuss the findings of emotional effects on wildlife conservation decision-making in the context of preference stability assumptions and present some conclusions and recommendations.

## Theoretical Background

### Emotions and their Measurement

In psychological theory an emotion is any mental experience with high intensity and a high degree of pleasure or displeasure (Cabanac 2002). Emotions involve different components: an informational component or stimulus, situation appraisal, physiological changes and action tendencies (Frijda 1986). The evaluation of the stimulus, the appraisal, needs criteria with which the emotional relevance of the stimulus is judged. These criteria are emotional dispositions. They can be innate or learned but are always present in individuals with a certain degree of abstraction. Therefore, emotional dispositions are relatively stable. On the other hand, emotional states (i.e. the emotional response to the stimulus) are temporary, and vary greatly in intensity from person to person and depending on the situation (Jacobs et al. 2012).

Four major categories of response systems are available in the literature to measure emotions: physiological measures, brain activity measures, behavioural measures, and self-reported measurements that allow capturing emotions by asking questions to respondents (Mauss and Robinson 2009). In this paper we adopt self-assessment measures, asking respondents about their emotional disposition and current emotional state. “Self-reports of emotion are likely to be more valid to the extent that they relate to currently experienced emotions” (Mauss and Robinson 2009, p. 213).

### Emotions in Decision-making

Emotions influence actual choices and behaviours (Lerner et al. 2015), in two main ways (Rick and Loewenstein 2008): anticipated emotions and immediate emotions. Individuals may anticipate their own future emotions and choose the outcome providing the highest positive emotion or emotions can be experienced immediately at the time of decision-making. When deciding, two types of emotions are experienced, incidental emotions and integral emotions (Lerner et al. 2015). Incidental emotions have nothing to do with the decision itself but arise from surrounding circumstances. For example, weather condition is likely to affect individual decision making because it has an influence on emotions (Schwarz 2012). Integral emotions on the other hand stem directly from the decision context, the emotions resulting from consideration of the decision or judgmental target itself. Integral emotions can be useful. For example, if a decision causes some anxiety or fear, this information can be a signal that we need to proceed with caution, being more risk-averse than risk-seeking (Västfjäll et al. 2016). Risk judgements are often made because of integral emotions (Loewenstein et al. 2001, Slovic and Västfjäll 2010). Integral emotions help categorize experiences along a good–bad dimension (Kahneman et al. 1997) and are readily accessible when making decisions, more than cognitions and all other emotions (Ortony et al. 1988).

### Emotions in Wildlife Conservation

Wildlife cause different emotions in individuals. Animals widely considered cute or beautiful tend to generate positive emotions such as happiness and joy, whereas animals considered dangerous and disgusting, tend to be more fearsome (Castillo-Huitrón et al. 2020). Fear towards predators has probably been genetically fixed throughout generations. It would be a defence mechanism against animals that represent a risk to human life, particularly large predators (Öhman 1986). Hence, humans have developed greater awareness toward potentially dangerous animals (LoBue and DeLoache 2008). Therefore, fear depends on an innate emotional disposition, which is automatically activated by a threat stimulus, such as the viewing of a wolf in the wild, in a video, or in a picture (Tappolet 2010).

The large set of emotions that arise in the context of wildlife may affect differently individual preferences for its conservation (Jacobs 2012). Positive emotions towards wildlife favour people’s attitude for supporting conservation actions (Gunnthorsdottir 2001). On the other hand, fear may generate attitudes and behaviours against the presence of predators or large carnivores (Cozzi et al. 2012, Røskaft et al. 2003, Zimmermann et al. 2001). Examples from around the world show that managing large carnivores is difficult when local communities are fearful. In such cases the popularity of conservation programs is low (Dickman 2010, Bath et al. 2008, Kaltenborn et al. 2006), wildlife management techniques are affected (Larson et al. 2015), and willingness to pay for conservation is low (Johansson et al. 2012). It is therefore clear how “society’s emotions toward wildlife may be key elements for decision-making on conservation issues” (Castillo-Huitrón et al. 2020, p.7).

Social media may potentially affect fear towards large carnivores, through the provision of extensive information on how predators are dangerous to people (Røskaft et al. 2003, Hughes et al. 2020). On the contrary, wildlife documentaries, spreading information about the ecological importance of animals and emphasizing positive appearances of wild animals (e.g. showing a cute bear cub or wolf pup), may promote positive emotions in people (Jones et al. 2019). Sometimes documentaries also ignore the threats posed by wildlife (Richards, 2013) and contribute to make people comfortable with potentially dangerous species. Conservation managers have noticed potential issues arising from visitors with highly positive attitudes towards carnivores (Soppe and Pershina 2019). Another worrisome aspect is that filming documentaries in natural areas allows close contacts between humans and wildlife and is increasingly habituating large carnivores to people (Herrero et al. 2005). Mass media representations can therefore provide negative or positive stimuli that may influence people’s emotions, attitudes and behaviour toward large carnivores’ conservation (Knight 2008, Wieczorek 2012). Thus, society’s preferences for large carnivores management may in some situations be based upon peoples’ emotional states emerging in response to media stimuli that overemphasize or underenphasize the dangers arising from human-wildlife cohabitation.

Even if some emotions, such as happiness, joy, satisfaction, and fear affect individual pro-ecological behaviours they are normally ignored in conservation studies, which sometime consider cognitive determinants of such behaviours (Tapia-Fonllem et al. 2010). However, since external stimuli may influence the level of fear towards wildlife, this study investigates the impact of visual stimuli on fear, preferences, and willingness to pay for wildlife conservation.

## Materials and Methods

### The Study Area

Data for this case study originated from a questionnaire survey administrated in Trentino, a mountainous province in the Northeast of the Italian Alps. Trentino is an important tourist destination, with around three million tourists per year and a good balance between winter and summer tourists. Overall summer tourists visit Trentino for nature-based reasons, such as walking, hiking, picnicking, and enjoying landscape, while winter tourists are mainly interested in skiing. In both the categories couples and families with children are most common. The incidence of Italian tourists is higher in summer (75%) than in winter (66%), and come mainly from neighbouring regions (P.A.T. Provincia Autonoma di Trento (2016)).

This area is important for nature conservation due to several rare and endangered species. The province includes one national park (Parco Nazionale dello Stelvio), two regional parks (Adamello-Brenta and Paneveggio Pale di San Martino) and several other Nature 2000 sites. Protected areas occupies more than one third of the total area (P.A.T. http://www.areeprotette.provincia.tn.it/). Among several interesting species, this study focuses on tourists’ preferences for conserving the wolf (*Canis lupus*), the lynx (*Linx linx*) and the golden alpine salamander (*Salamandra atra aurorae*), a rare subspecies of the alpine salamander. The wolf and the lynx became extinct in Trentino towards the end of the 19th century and have since naturally recolonised, the wolf from the Italian Apennines and the lynx from Switzerland. At the time of the survey, there were just seven wolves and one lynx in the regional area, therefore the population size was not enough to assure the survival of the species. The golden alpine salamander is a rare amphibian living only in a limited area of the province with a population of about ten specimens.

### Survey Design and Administration

Face-to-face questionnaires were administered to tourists, in the summer of 2015. Tourists were intercepted in different natural areas, chosen to represent the entire province.We surveyed tourists because they are the direct users of natural areas and may be in contact with wildlife during their outdoor activities.

Interviewers asked every second tourist to participate to the survey. A face-to-face survey following NOAA panel recommendations (Arrow et al. 1993) was chosen as the most convenient method to collect tourist stated preferences. In fact, a database with a registered contact list of tourists is not available to be considered as an alternative sampling method. Face-to-face questionnaires are advantageous to encourage respondents’ concentration and effort. However, there are some limitations to consider, for example potential experimenter demand effects in the treatments (De Quidt et al. 2019). Experimenter demand effects may occur, for example, if respondents considered the picture of a wolf a cue about what interviewers expect as an answer. To limit this effect interviewers were instructed to minimize social interactions with respondents. Questionnaires were filled-in by respondents themselves, while interviewers only explained the different sections of the questionnaire and provided assistance on how to fill out choice cards. This is one of the measures suggested to limit experimenter demand effects, which should not represent a serious concern in this study. The average completion time was around 15 min.

The questionnaire was designed following the guidelines for DCE available in the literature (Riera et al. 2012). It was composed by 34 questions, organized in three thematic sections. The first part of the questionnaire included a question about participating in outdoor activities, questions on value orientations^2^ and emotions towards wildlife. The second section contained the choice cards, preceded by some text read by interviewers that contained information on the current status of the animals, the content of the cards and the way to answer questions. The target population size to ensure viable animal populations were not explained in order not to affect personal choices. Respondents were informed that money raised from the tickets proposed in the choice cards would have been used to fund the restoration of a viable population of the animals. A consequentiality script was also included (Carson and Groves 2007), which informed respondents that results could be used for policy. They were therefore asked to complete choice tasks with commitment and thinking as if they actually had to pay the amounts of chosen alternatives. The last section contained socio-demographic questions. A pre-test of the questionnaire was conducted in June 2015 on a sample of 63 tourists, to check wording and collect priors, which were used to generate a D-efficient Bayesian design for the main survey (Bliemer et al. 2008). Respondents were asked to complete 12 choice tasks, each of which was composed of three alternatives (two efficiently designed alternatives and a null alternative). The answer format was the Best-Worst, in which respondents choose the most preferred alternative among all the alternatives offered in the choice card and the least preferred over the remaining alternatives (i.e. the original set of alternatives less the preferred alternative). The Best-Worst format allows collecting a larger number of observations compared to the traditional pick-one alternative, with only a small increase in the effort for respondents (Louviere et al. 2013).

Non-monetary attributes were the number of animals for wolves, lynx, and salamanders. Levels were decided after focus groups with experts of wildlife management. They stated that 40-50 specimens would assure a viable population for wolf and lynx and 90 would be the maximum regional carrying capacity. The levels were therefore 0, 15, 30, 45, 60, 75, and 90 individual animals for each species. We recognize that wolves and lynx might compete for food and territory and therefore correlation might occur between these attributes. However, given the size of the study area and the relatively small number of each animal as well as the differences in their preferred prey species, experts feel that any conflict is likely to be small. In addition, we did not propose management measures to obtain a given size of population, therefore correlation between attributes should not be a problem for the experiment.^3^ The monetary attribute was the price of a ticket to visit protected areas in the region, that ranges from 0 to 18 € with seven levels. At present, local parks charge no fees but increasing financial requirements to co-finance wildlife management activities may lead to the introduction of a visitor ticket. An alternative to the WTP approach, which estimates the benefit of wildlife in terms of a welfare gain from the current situation, is the Willingness to Accept (WTA) approach. In a WTA study, the welfare change of wildlife is estimated in terms of restoration from a former condition (Brown and Gregory 1999). Therefore, the WTA approach is generally suitable to address restoration projects, as it addresses the avoided loss of welfare caused by extinction. As WTA measures are usually larger than WTP, the welfare change of wildlife may be under-estimated if WTP is adopted (Sayman and Öncüler 2005). In Trentino, the extinction of wolves and lynx occurred in the mid-1800. Therefore, the loss of welfare caused by extinction has not been experience from the current generations of visitors and the restoration may be treated as a gain from the current situation. For this reason, it was established that a WTP study was more appropriate than WTA due to the particular situation of the study area.

The impact of external *stimuli* on the level of fear and on preferences and willingness to pays to protect wildlife was explored with two treatments. Each treatment was randomly assigned to one fourth of the total sample. The other half of respondents received no treatments and acted as a control group. We assigned the first treatment to 105 respondents, the second treatment to another 105 respondents and the control group was composed of a further 210 individuals. Treated participants were first asked to self-assess their emotional dispositions when thinking about wolves, lynx and salamanders. Respondents rated their level of fear, joy, anger, disgust, sadness and pleasure on a 7-Point Likert-type scale from “Not at all” to “Very strong”, answering this question for each animal species: “Six emotions are listed below. For each emotion please indicate the extent to which the … (name of the animal) evokes this emotion in you”. Subsequently, they were shown pictures of a lynx, a salamander and a wolf, and asked to self-assess their emotional state after each picture, on a similar 7-Point Likert-type scale question with this wording “Six emotions are listed below. For each emotion please indicate the extent to which this photo of a … (name of the animal) evokes this emotion in you”. Therefore, respondents answered first the questions on emotional dispositions, then looked at the pictures and answered the question on emotional states for each animal. They were not given the possibility to go back to questions they answered, moving from states back to dispositions, and change their answers.

The pictures remained visible for the entire length of the choice task and were included in the choice cards. No information was provided together with the pictures. The two treatments only differed in the picture of the wolf that respondents were exposed to. The first group, which we refer to as the fear-treated group, received a picture of a snarling wolf. The second group, which we conversely called the assure-treated group, received a picture of a cheerful wolf. The pictures of the lynx and salamander were the same across treatments. The control group received no pictures of animals on the page preceding the choice cards and similar choice cards than the treated groups but with a picture of a neutral wolf instead of a snarling or cheerful wolf on them.

**Fig. 1:**
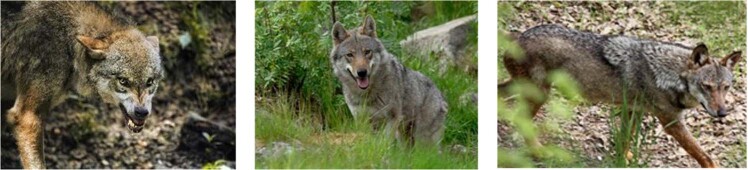
Pictures used for the fear (left) treatment, the assuring (centre) treatment and the control (right)

The pictures were tested in focus groups, where the pictures more suited to represent a reassuring, a scary, and a neutral wolf were selected by focus groups participants. Pictures used for the treatments are reported in Fig. 1.

Pictures were selected as a treatment vehicle because they are common in psychological and medical studies (e.g., Heinberg and Thompson 1995, Hofer et al. 2006, Schneider et al. 1994, Wadlinger and Isaacowitz 2006, Wang et al. 2005). In addition, pictures have been previously adopted for wildlife, for example with respect to wildlife preferences of zoo visitors (Frynta et al. 2013, Marešová et al. 2009).

### Econometric Analysis

Our modelling approach is grounded on the Random Utility Theory (Manski 1977), for which the utility of respondent *n* for the alternative *i* in the choice situation *t* may be described by the following utility function, linear in the parameters:

1 $$U_{int} = \beta X_{int} + \varepsilon _{int}$$

where *β* is a vector of parameters to be estimated, *X*_*int*_ a vector of attributes that describe alternative *i*.

In our study we suspected a non-linear relationship between WTP and population size, because people might be willing to contribute for conservation but, at the same time, they do not want too many animals. To account for this non-linearity, we compared a dummy coding for each attribute level and a quadratic specification of the utility function. The level of log-likelihood was similar in the two models, while the Bayesian Information Criterion (BIC) was smaller for the utility function specified in quadratic terms, which was then selected as preferred model specification.

Since the utility function for population sizes of the three animals includes quadratic terms, the deterministic component of the utility associated with wolf population takes the form:

2 $$U_{int} = \beta _1N\_wolf_{int} + \beta _2N\_wolf_{int}^2$$

where *N*_*wolf* is the level that indicates the number of wolves in alternative *i* in choice situation *t*. This leads to a parabolic-shaped utility function, whose vertex represents the population size with highest or lowest utility for respondents, depending on the signs of the coefficients. If the utility increases up to a given population size and then decreases, this parabolic utility must open downward and reach a top in correspondence of a positive number of animals. Therefore, we anticipate a positive *β*_1_ and a negative *β*_2_ for all treatment groups.

Assuming that the error term is i.i.d. extreme value type I distributed, a certain sequence of choices can be modelled with a conditional logit model (MNL), whose probabilities can be calculated as follows (McFadden, 1973):

3 $$\Pr \left( {i_{nt}\left| {x_{int}} \right.,\beta } \right) = \mathop {\prod}\nolimits_{t = 1}^{T_n} {\frac{{e^{\beta ^{\prime}_nX_{ni}}}}{{\mathop {\sum }\nolimits_j e^{\beta ^{\prime}_nX_{ni}}}}}$$

It is well-known that the standard MNL has the limitation of providing a point estimate for each coefficient, which is equivalent to assuming preference and scale homogeneity for the entire sample. Such a condition is unlikely to hold, therefore analysts are often concerned in estimating more flexible models that account for taste heterogeneity and scale heterogeneity (Train 2009). In this regard, the generalized mixed logit model is used (Fiebig et al. 2010, Hensher et al. 2015). This model assumes that coefficients are individual-specific and follow a random distribution, for which a location and a scale parameter is estimated:

4 $$\Pr \left( {i_{nt}\left| {x_{int}} \right.,\beta } \right) = {\int} {\frac{{e^{\beta ^{\prime}_nX_{ni}}}}{{\mathop {\sum }\nolimits_j e^{\beta ^{\prime}_nX_{ni}}}}} \varphi \left( {\beta \left| b \right.,\,{\Omega}} \right)d\beta$$

In which:

5 $$\beta _n = \sigma _n\left[ {\beta + {{{\mathrm{{\Delta}}}}}z_n} \right] + \left[ {\gamma + \sigma _n\left( {1 - \gamma } \right)} \right]{{{\mathrm{{\Gamma}}}}}v_n,$$

where:

Z_n_ = a set of M characteristics of individual n that influence the mean of the taste parameters;

v_n_ = a vector of K random variables with zero means and known (usually unit) variances and zero covariances;

*σ*_*n*_ = the scale factor of the idiosyncratic error term that varies among individuals. It is assumed to be log normal distributed with mean $\bar \sigma$ and standard deviation *τ*; *τ* is the key parameter that captures the unobserved scale heterogeneity;

*γ* = a weighting parameter that indicates how variance in residual preference heterogeneity varies with scale in a model that includes both. It expresses the relative importance of the overall scaling of the utility function *σ*_*n*_, as opposed to the scaling of the individual preference weights;

X_ni_ = the K attributes of alternative *i* in choice situation *t* faced by individual *n*;

$\varphi \left( {\beta \left| b \right.,\,{\Omega}} \right)$ is the probability density function of the distribution of the coefficients.

In environmental applications, it is common practice to assume normally distributed coefficients. In our Best-Worst format respondents are asked to state their most (best) and least (worst) preferred alternatives in a set of three alternatives *i* in each of the twelve choice tasks *t*. We assume that each respondent choose his/her most preferred alternative in each choice tasks containing three alternatives (i_1_, i_2_, i_3_) and subsequently the worst alternative, selected among the remaining two because the previous selection of the first alternative reduces the choice set by one. The non-chosen alternative represents the second-best alternative. As the Best-Worst approach allows us to retain two choice-observations from each choice task we estimated our models by using the “exploded” parametric mixed logit model (Luce and Suppes 1965, Scarpa et al. 2011), whose probabilities are computed as the product between the probability of the best choice and that of the second best:

6 $$Pr[ranking\,i_1,i_2,i_3] = {\int} {\frac{{e^{\beta ^{\prime}_nX_{ni_1}}}}{{\mathop {\sum }\nolimits_{i = i_1,i_2,i_3} e^{\beta ^{\prime}_nX_{nij}}}} \times \frac{{e^{\beta ^{\prime}_nX_{ni_2}}}}{{\mathop {\sum }\nolimits_{i = i_1,\,i_2} e^{\beta ^{\prime}_nX_{ni}}}}} \varphi \left( {\beta \left| b \right.,\,{\Omega}} \right)d\beta$$

In our model we also account for differences in the scale factor between the Best and the Worst choice through an extension of the scale parameter *τ*, as follows: $\tau = \tau + \eta d_s$, where *η* is a choice specific scale parameter and *d*_*s*_ = 1 for the Worst choice and zero for the Best choice. We therefore captured both scale differences across choices due to changes in choice set composition and data-specific scale heterogeneity effects.

To test if the two treatments lead to differences in observed choices, each parameter was interacted with a dummy representing being exposed to the scary or reassuring picture of the wolf, relative to the neutral treatment. To test if the effect of the picture is maintained along the choice process, the parameters for wolf were interacted with the number of the choice card.

The welfare analysis that follows model estimation is conducted in terms of the marginal rate of substitution between non-monetary and monetary attributes, which returns WTP measures that indicate the amount of individual income that respondents are willing to sacrifice in exchange for larger populations of wolf, lynx and salamanders in Trentino. In the presence of a quadratic utility function, the willingness to pay is calculated as follows:

7 $$WTP_J = - \frac{{N_j(\beta _1 + \beta _2)}}{{\beta _{cost}}}$$

where *N*_*j*_ is the population size for the *j-th* animal species, while *β*_1_ and *β*_2_ refers to the linear and quadratic coefficients.

In the generalized mixed logit model, assuming a normally distributed coefficient for the cost attribute complicates the estimation of the WTP, because it would lead to a ratio between two normal distributions, with no finite central moments. For this reason, we assumed a fixed cost coefficient to allow WTP estimation. We estimated WTP for different size animal populations using the Krinsky-Robb procedure with 5,000 draws (Krinsky and Robb 1986).

## Results

We asked 651 tourists to participate, out of which 420 completed questionnaires, yielding a response rate of 65%. Respondents were on average 43 years old and females accounted for the 53.3% of the sample (males constituted the remaining 46.7%). Most respondents had a high school degree (41%), while the share of respondents with a university degree was around 37%. The median annual net income bracket was €10-20 thousands. A large portion of respondents come from neighbouring regions, Veneto, Lombardy and Emilia-Romagna. Descriptive statistics of the sample are comparable to the average regional tourists described in official reports (P.A.T. Provincia Autonoma di Trento (2016)).

### Emotional Responses

**Fig. 2:**
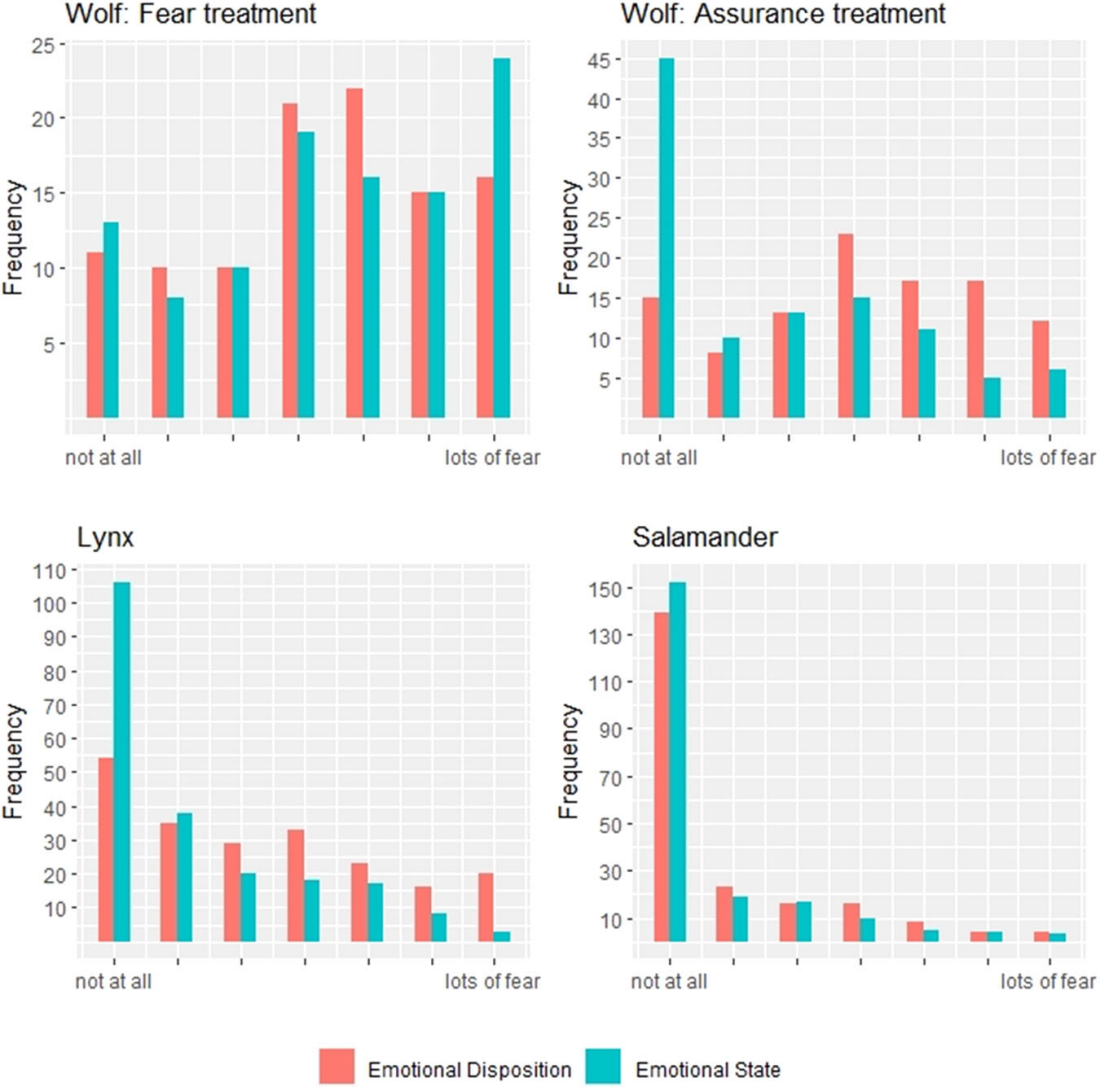
Fear toward wolves, lynx and salamanders: emotional disposition and state

Self-assessed levels of emotional disposition and emotional state towards wolves, lynx and salamanders are reported in the bar chart in Fig. 2.

With respect to wolves, respondents in the two treatments report broadly comparable average levels of emotional disposition on a 0–6 scale; the average score reported in the fear treatment is 3.35, while the average score in the assurance treatment is 3.12. Both answer distributions are left-skewed, with scores relatively concentrated around the values of 3 and 4 and a *t* test on the means of the two treatments does not find a statistically significant difference (*p* value = 0.3788). The treatment administration impacts the two treatments differently. In the fear treatment, the frequency of answers to emotional states mildly shifts towards ‘strong fear’ but the overall distribution is comparable to the distribution of emotional dispositions. This is also reflected in similar average scores (3.35 before treatment and 3.50 after treatment) that the t test presented in Table 1 does not find to be statistically significant (*p* value = 0.50). In the assurance treatment, fear levels after the treatment are considerably lower. The distribution is strongly skewed to the right, with a share of respondents who reports ‘no fear’ (value of zero) in excess of 40% and mean values before and after the treatment that are statistically different (*p* value < 0.0001). The *t* test that compares average scores of emotional states between treatments is statistically significant (*p* value < 0.0001).

**Table 1:** *T* tests on fear toward wolves, lynx and salamanders: emotional disposition and state

Wolves	Fear treatment		Assurance treatment		
	Mean	Std.dev.	Mean	Std.dev.	*P* value*
Disposition	3.35	1.86	3.12	1.89	0.3788
State	3.50	2.02	1.77	1.92	<0.0001
*P* value^*^	0.50		<0.0001		
Tests of fear towards Lynx and Salamander					
	Disposition		State		
Species	Mean	St. dev.	Mean	St. dev.	*P* value*
Lynx	2.30	1.98	1.23	1.61	<0.0001
Salamander	0.85	1.47	0.67	1.33	0.186

^*^T tests for the comparison of the means

Moving to lynx and salamanders, the initial emotional disposition is lower for both animal species compared to wolves and frequency distributions are both right-skewed. The picture of the lynx contributes to lower the average fear to a statistically significant extent (*p* vale < 0.0001), whereas the fear of salamander is not significantly altered following picture display (*p* value = 0.186).

Personal characteristics that affect answers to emotional dispositions and emotional states are explored using ordered logit models, available in Table 2. Emotional dispositions and emotional states are pooled together, so that the dataset comprised a panel of 2 observations per respondents.^4^

**Table 2:** Ordered logit models for emotional dispositions and states

	Wolf		Lynx		Salamander	
	Coefficient	*t* value	Coefficient	*t* value	Coefficient	*t* value
Emotional state vs disposition towards the species (fear treatment)	0.292	1.369	−1.153***	−6.243	−0.353***	−1.621
Emotional state vs disposition towards the species (assurance treatment)	−1.541***	−6.697				
Female	0.867***	4.498	0.841***	4.171	1.276***	4.859
Age class (ref. 18–24)						
25–34	−0.332	−1.080	−0.535*	−1.715	−0.781*	−2.058
35–54	−0.656**	−2.026	−0.911***	−2.730	−0.9***	−2.264
55–64	−0.542	−1.257	−0.413	−0.970	−0.886	−1.643
65 and older	0.55	1.055	−0.499	−0.907	−0.208	−0.332
Educational attainment (ref. Primary school)						
Middle school	0.512	1.116	0.311	0.662	1.111	2.048
High school	1.041***	2.702	0.72*	1.855	0.148*	0.328
University degree	0.745*	1.874	0.692*	1.711	0.345*	0.732
Doctoral studies	1.236**	2.392	1.215**	2.341	1.636**	2.685
Env	−0.243	−0.666	−0.215	−0.580	−0.292	−0.675
Income level (ref. (less than 9.999 €/year))						
10K–20K €/year	0.008	0.028	0.706**	2.547	1.078**	3.222
20K–30K €/year	−0.054	−0.190	0.424	1.417	0.217	0.551
30K–40K €/year	0.611	1.533	0.922**	2.251	1.489**	2.918
40K–60K €/year	−1.559***	−3.602	−0.368	−0.825	0.436	0.792
80K–100K €/year	0.063	0.086	0.706	0.915	1.35	1.569
>100 K €/year	2.756***	2.808	1.972**	2.410	0.142**	0.115
Cut-off 1	−1.132***	−2.578	−0.286	−0.645	1.623	3.115
Cut-off 2	−0.542	−1.242	0.548	1.235	2.218	4.218
Cut-off 3	0.063	0.144	1.132**	2.546	2.864**	5.352
Cut-off 4	0.984**	2.246	1.847***	4.111	3.663***	6.601
Cut-off 5	1.833***	4.132	2.651***	5.737	4.353***	7.449
Cut-off 6	2.718***	5.981	3.479***	7.141	5.149***	7.965
LL	−740.82		−679.94		−433.67	
AIC	1529		210		913	
Respondents	210		904		210	

Robust standard errors were clustered at individual level

Regarding wolves, the variables labelled “emotional state” captures answers to emotional state questions compared to the baseline of emotional dispositions. Consistently with t tests, these variables are statistically significant and negative for the subsample of the assurance treatment, while it is not significant for the subsample of fear-treated individuals. Considering gender, females are more likely to state larger levels of fear. The coefficients associated with age cohorts are negative, which indicates that younger people are less likely to fear wolves compared to the baseline respondents of 18–24 years of age. The exception is the bracket of respondents older than 65, who are associated with a positive coefficient but it is not statistically significant. Educational classes have positive coefficients, thus the reference level of respondents with a primary education is less likely to state large fear compared to more educated respondents. Members of environmental associations have a negative coefficient but it is not statistically significant. With respect to individual financial situation, a systematic pattern based on increasing income is not detected as only two income levels are associated with a significant coefficient. Results for lynx and salamanders are broadly consistent with results for wolves, in fact significant coefficients never change sign across models and only magnitude and significance differ.

### Choice Model

Results of the generalized mixed logit model for emotional states are provided in Table 3.

**Table 3:** Results of the generalized mixed logit model

Attributes	Mean coefficient	Standard error	Std. dev. Coeff.	Standard error
Control group (main effects) coefficient				
Wolf	0.05671***	0.00590	0.01134***	0.00182
Wolf^2^	−0.00060***	0.00005	0.000091	0.00003
Lynx	0.05453***	0.00868	0.00977***	0.00140
Linx^2^	−0.00056***	0.00009	0.000003	0.00004
Salamander	0.02418***	0.00749	0.01642***	0.00170
Salamander^2^	−0.00020***	0.00007	0.00001	0.00004
Null alt.	−8.25057***	0.95943	4.57247***	0.46812
Cost	−0.08450***	0.00997		
Interactions with the fear group treatment				
Wolf	0.02201**	0.01034	0.01910***	0.00465
Wolf^2^	−0.00021**	0.00008	0.00007	0.00006
Lynx	0.01398	0.01385	0.01766***	0.00363
Linx^2^	−0.00011	0.00014	0.00008	0.00008
Salamander	0.00420	0.01198	0.00837	0.00706
Salamander^2^	−0.50206	0.00014	0.00011	0.00007
Null alt.	1.48852	1.08426	5.52328***	0.96200
Cost	−0.01535	0.01717		
Interactions with the assuring group treatment				
Wolf	0.04381***	0.01209	0.00078	0.00501
Wolf^2^	−0.00032***	0.00010	0.00005	0.00009
Lynx	0.02700*	0.01623	0.00126	0.00781
Linx^2^	−0.00024	0.00016	0.00003*	0.00009
Salamander	−0.00723	0.01469	0.02187***	0.00488
Salamander^2^	0.00004	0.00014	0.00001	0.00007
Null alt.	−0.00127	1.35252	1.10300	0.67288
Cost	−0.06279***	0.02084		
Interaction with the number of choice card				
Wolf_Fear	−0.00077	0.00055	0.00103	0.00066
Wolf_Assure	−0.0004	0.00063	0.00145*	0.00077
Random scale parameters				
* τ* Scale	0.16954***	0.03090		
* τ* BW	0.99257***	0.09452		
_* γ*_	2.11345***	0.42855		
* σ*	0.98974***	0.34106		
Obs	10,080			
Respondents	420			
log_L	−3658.38			
McFadden’s R2	0.669			

Wolf^2^, Lynx^2^ and Salamander^2^ refer to the squared number of individuals for each species

** and *** indicate significance levels at 5% and 1%, respectively

As expected from economic theory, the cost coefficient is negative for the control group and the two treatments, suggesting decreasing marginal utility at higher price levels. However, it is not statistically significant for the fear-treated group, meaning that the fear group does not have a statistically different marginal utility of money than the control group. All coefficients associated with animal species are positive for the linearly coded number of animals and negative for the squared coefficient. The negative coefficient for the quadratic coding suggests that utility decreases for large populations. This result indicates that people could be willing to pay to restore viable populations but they do not want too many specimens, possibly due to concerns over environmental pressures and social conflicts. Salamanders have the smallest contribution to the utility function of tourists, as their coefficients are the lowest. The null alternative coefficient, representing utility for the scenario with zero animals, is negative, which indicates that respondents are on average better-off in the presence of wolves, lynx and salamanders. Most of the standard deviations for population sizes are statistically significant, which is an indication that sample preferences are heterogeneous.

With respect to interactions with the fear treatment, the coefficients of the wolf population have the same sign of the control group, which indicates that the utility associated with wolves has a similar bell-shaped trend. The non-significant coefficients for lynx and salamander indicate no cross-species effects of the treatment. The null alternative coefficient is non-significant, which indicates that the treatment did not significantly affect respondents’ utility. Standard deviations are statistically significant for the coefficients associated with wolf and lynx, suggesting preference heterogeneity across the sample.

In the interactions for the assure-treated group, the coefficient for the number of wolves is positive and twice the coefficient of the interaction for the fear-treated group, suggesting higher utility, whereas coefficients for lynxes and salamanders are not different from zero. The standard deviation is significant for the coefficient of salamander population, whereas it is not different from zero for wolf and lynx.

The two parameter interactions introduced to understand if the effect of the pictures is maintained during the tasks in the two treatments (*Wolf_Fear* and *Wolf_Assure*) are not statistically significant, which indicates that the treatment effect provided by pictures does not decrease during the choice exercise.

Finally, our results show the presence of scale heterogeneity across individuals, in fact the coefficient labelled $\tau$ *Scale* is positive and highly significant. The scale parameter associated with changes in choice set composition ($\tau$ *BW*) is also positive and significant at the 1% level, indicating that the Best choice has lower uncertainty compared to the Worst choice.

### WTP for the Conservation of Wolves, Lynx and Salamanders

**Fig. 3:**
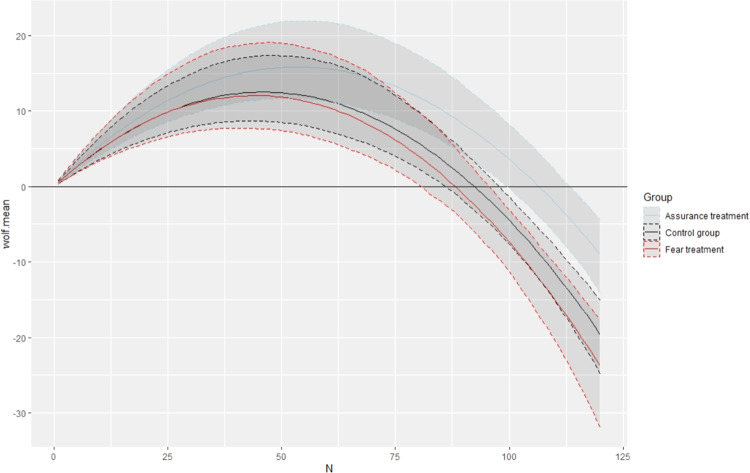
WTPs for conserving wolves in fear and assuring treatments and control group

Figure 3 shows WTPs for wolf populations of 1-120 size, estimated for the two treated groups and the control. The trend is similar, as the WTP increases for larger population sizes up to 50 specimens and then it decreases. WTPs for a population of 50 wolves, 50 lynx and 50 salamanders are reproduced in Table 4.

**Table 4:** WTP for a population of 50 and 90 specimen across treatments

	WTP for a population of 50			WTP for a population of 90		
	Control Group	Fear Group	Assurance group	Control Group	Fear Group	Assurance group
Wolf	12.16	11.98	15.97	1.07	−0.88	8.22
	(8.21–17.08)	(8.25–18.16)	(11.42–22.16)	(−2.09 to 4.59)	(−4.94 to 3.67)	(4.30–13.07)
Lynx	12.41	14.2	11.99	3.34	7.56	4.73
	(8.36–17.63)	(9.07–21.75)	(7.70–17.91)	(0.96 to 6.29)	(3.98–12.60)	(1.83–8.37)
Salamander	5.18	4.15	0.61	3.41	1.21	0.95
	(1.96–9.02)	(0.18–9.33)	(−2.24 to 4.46)	(0.10 to 7.71)	(0.18–9.33)	(−2.84 to 5.34)

95% Krinsky-Robb confidence interval in parenthesis

It can be noticed that average WTPs for population sizes over 50 specimens in the assure-treated group are systematically larger than the control group, and WTPs of the fear-treated group are lower than both the assure-treated and the control groups. Differences become larger as the population size increases. At a population of 90 wolves (Table 4), a Poe test conducted on the empirical distributions of the WTP returns statistically different WTPs between the fear treatment and the assurance treatment (p value = 0.007). The test returned also statistically significant differences between the assurance treatment and the control group (p value = 0.013), while WTPs in the control and the fear treatment for 90 wolves were not statistically different (p value = 0.27).

## Discussion

The two treatments had different effects on people’s self-rated emotions. Compared to the initial emotional disposition, the fear treatment did not significantly change the self-rated level of fear towards wolves, while the assuring treatment decreased fear levels. A possible explanation for this result is that fear towards wolves is a deep emotion and the picture of a scary wolf just fixed that deep emotion. In contrast, a smiling wolf that resembles a pet dog lowered the level of fear toward wolves. There is a dearth of papers that manipulate fear versus assurance to validate our findings but a pioneering study by Mewborn and Rogers (1979), undertaken using videos as treatments and physiological and self-rated measures as response variables, found comparable results. In their study, the fear stimulus was not associated with attitudinal change, whereas the reassurance stimulus facilitated attitude change.

The DCE indicates that tourists in Trentino are willing to financially contribute to the conservation of wolves, lynx, and, to a much lower extent, salamanders. Overall, these results confirm previous research, which suggests that people are more in favour of charismatic species and mammals conservation than reptiles (Colléony et al. 2017, Martín-López et al. 2008). The maximum WTP for wolves is estimated for a population of 50 individuals. Larger populations are less favourable probably due to potential dangers to public safety. The number of 50 wolves might have been considered as a safety threshold from our respondents given the size of the regional area. Experts estimate a regional viable population of about 40-50 wolves and a maximum regional carrying capacity at about 90, therefore people’s preferences meet regional wolf conservation requirements. The three groups had comparable WTP for a population of 50 wolves and differences become statistically relevant at population sizes larger than 90, which indicates that the impact of treatments affect preferences in the presence of very large populations. These results suggest that for a small number of wolves people are generally in favour of conservation regardless of the treatment, whereas above a certain threshold the emotional state affects WTP and respondents with fearful emotional state are less willing to pay than respondents with a lower fearful emotional state. The increasing influence of emotional states on preferences for wolf conservation at large populations may be explained by the increasing chances of an encounter during outdoor activities.

Wildlife conservation is a public good with direct consequences on tourists’ recreational experience. If tourists were afraid that increasing wolf populations jeopardizes safety while hiking in the woods, they might oppose conservation. People always own some degree of fear, as it is innate, which translates into stable preferences for wildlife conservation. But this level can be altered by external stimuli, for example descriptions portrayed by media. Media influence and media effects are topics well studied in the literature (Potter 2012). Mass media can produce positive or negative stimuli that affect thoughts, attitudes, emotions and behaviour (Jones et al. 2019, Hughes et al. 2020). However, the induced emotional state is not stable but context-dependent and likely does not affect long-term preferences. It can result in a contingent influence on stated preferences and potentially impose a threat to the validity and the reliability of stated preference estimates. At present, the effect of fear on preferences and WTP caused by our treatments is small and most evident for large populations and the assure-treated group. This can be explained by the fact that not all external stimuli result in sharp changes; some stimuli can simply reinforce existing beliefs, as seems to have happened in our fear treatment.

The assure-treated group returned results that converge to previous psychological studies, which suggest that people with pleasant feeling about something perceive higher benefits and lower risks (Slovic et al. 2004). The fear treatment had a lower-than-expected impact on stated choices and was only statistically significant for population sizes over 50. This result partially confirms Johansson et al. (2012) that found that fear for large carnivores was negatively associated with WTP for conservation policies, since our result depends on population size.

Overall, our findings suggest concerns due to respondents’ emotional state of fear for the validity and reliability of stated preferences. The effect is particularly evident for the assurance stimulus, whereas it is less clear with respect to the fearful stimulus. Relevant impact of emotions on stated choices have been found previously by Notaro et al. (2019), Araña and León (2009), Araña and León (2008) and Araña et al. (2008), hence the merit of research in emotional states and stated preferences, which is still limited despite the behavioural literature indicating that emotions influence all situations of individual choices (Lerner et al. 2015). Some studies found no effect of emotions on stated preferences, for example the paper by Hanley et al. (2017), who investigated sadness and happiness on preferences for beach recreation. This mixed evidence in the literature suggests that more research is recommended to draw meaningful conclusions.

The study of fear as emotional state is particularly relevant in highly emotional situations such as wildlife conservation. If people make choices according to their emotional state of fear an element of context-dependence is introduced in the survey. In this work the external stimuli altered the level of fear towards wildlife. Respondents with fearful emotional state are willing to pay less to protect wolves than respondents with lower fearful emotional state. When stated preferences towards wildlife are affected by the emotional state of fear due to contextual external stimuli, welfare analysis is not grounded on stable preferences and may lead to sub-optimal conservation policies. The relevance of this issue is greater in situations where a wildlife-related topic is highly emphasized, positively or negatively, by social networks, mass media and opinion leaders. The case of context-dependent choices requires further efforts in stated preference surveys, whose design should encourage respondents to make choices in neutral settings.

This study had some limitations that should be considered to integrate and improve future research. An important issue to take into consideration in our study is whether aggregated WTPs would be affected by the financial position of our respondents. The demand for biodiversity conservation and the capacity to meet this demand increase with a nation’s wealth (Jacobsen and Hanley 2009), as the social WTP for environmental goods depends on the level of income and its distribution (Breffle et al. 2015, Baumgärtner et al. 2017). In the literature equity adjustment factors have been proposed to correct welfare measures estimated from less wealthy people because of income constrains, ranging from around 13% (Breffle et al. 2015) to 16% (Baumgärtner et al. 2017). Their value depends “on the (in-) equality preferences of society” (Baumgärtner et al. 2017, p.50). Therefore, considering income inequalities, our WTPs could be slightly underestimated. Future studies should be designed in order to estimate a site- specific equity adjustment factor. A second limitation is the relatively small sample size of the treatments, which may be increased to obtain more robust estimates. Then, the occurrence of experimenter demand effects is another potential limitation in this study, although these effects are smaller with field surveys than lab surveys. While some degree of experimenter demand effects can never be ruled out, in this study they were limited by minimal interactions between interviewers and respondents and by means of pictures for all animals, not just the wolf, to avoid respondents believing that the focus of our study was a particular species. To further reduce experimenter demand effects future research may be conducted online, completely eliminating interactions with the interviewer.

## Conclusions

The number of large carnivores is often related to the feelings people have for these wild animals as these feelings turn into social and political pressures for their conservation (Trouwborst et al. 2017). Given that integral emotional dispositions are relatively stable, they might affect preferences for wildlife conservation, which are also relatively stable. When this is the case, stated preference methods allow eliciting stable preferences. Conversely, integral emotional states are not stable but context-dependent, which may lead to biased welfare estimates, thereby communicating incorrect policy recommendations to policy makers and wildlife managers.

This study investigated the effect of fear as emotional state on tourists’ stated preferences for wildlife conservation and found varying results associated to the two proposed treatments. The impact of an assuring picture contributed to lower fear and increase WTP for carnivores. The impact of a fearful picture was smaller and only limited to large population of wolves exceeding 50 specimens. Overall, the balance of evidence suggests that emotional states may influence preference stability for wildlife, especially for large populations. Emotional states may be altered by external stimuli, e.g., mass communications. These results can be explained by the stream of behavioural literature for which emotions affect the higher levels of cognitive processes and related decision-making. In stated preference studies, severe manipulation of the emotions may lead to context-dependent choices and biased WTP estimates. Therefore, it is advised to investigate the decision context during surveys and take it into consideration when reporting the results, especially in highly emotional situations such as wildlife management and conservation. When feasible, another recommendation is to avoid survey administration in times where emotions are potentially high (e.g., soon after a wildlife-related incident, such as a wolf killing or injuring a human). The empirical research on emotions is still limited and stated preferences could be affected in several different ways by different emotions, we therefore encourage further studies on this important topic.

From a policy perspective, this study provides important implications for wildlife decision-makers. There is evidence that viable populations of wolves and lynx are welfare-increasing for tourists, and provide the maximum welfare compared to lower or larger population of species. Conservation decisions may not be based on user preferences exclusively; the ecological stability of the environment is probably the main driver. However not including the human dimension when designing conservation policy may lead to conflicts among stakeholders (Drouilly and O’Riain 2021). The welfare implications of this work are a further evidence of the benefits of wildlife conservation, which may reconcile socio-economic and environmental objectives, provided that effective communication strategies are put in place (Miller et al. 2019).

## Footnotes

1 The compensatory decision rule consists in trading-off all attributes to determine the alternative with the highest utility.

2 The effect of tourists’ value orientations on preferences and WTP is examined in Grilli et al. (2018) while policy implications of conservation in Notaro and Grilli (2021).

3 Correlation might occur if, for example, we proposed to increase the size of population through an improvement of the habitat quality. This would cause an increase in the population of the three animals simultaneously. Avoiding this allowed us to have uncorrelated levels for the populations and the relative importance of the three animals for respondents is reflected in the stated WTP.

4 Panel models were not appropriate for this analysis because independent variables do not vary within groups, i.e. respondents, and would be dropped due to collinearity. For this reason, a pooled ordered logit model was implemented and standard errors were clustered at individual level, to account for the fact that observations from the same respondent are related.
